# Entropic Density Functional Theory

**DOI:** 10.3390/e26010010

**Published:** 2023-12-21

**Authors:** Ahmad Yousefi, Ariel Caticha

**Affiliations:** Department of Physics, University at Albany, Albany, NY 12222, USA

**Keywords:** density functional theory, Hohenberg–Kohn theorem, entropic inference, method of maximum entropy, inhomogeneous fluids

## Abstract

A formulation of density functional theory (DFT) is constructed as an application of the method of maximum entropy for an inhomogeneous fluid in thermal equilibrium. The use of entropy as a systematic method to generate optimal approximations is extended from the classical to the quantum domain. This process introduces a family of trial density operators that are parameterized by the particle density. The optimal density operator is that which maximizes the quantum entropy relative to the exact canonical density operator. This approach reproduces the variational principle of DFT and allows a simple proof of the Hohenberg–Kohn theorem at finite temperature. Finally, as an illustration, we discuss the Kohn–Sham approximation scheme at finite temperature.

## 1. Introduction

Density functional theory (DFT) is one of the most widely used methods for calculations of the structure of inhomogeneous many-body systems including atoms, molecules, liquids, solids, and surfaces [1,2] (for a pedagogic introduction, see [3]). The theory, which finds its earliest roots in the Thomas–Fermi–Dirac model, was first introduced in its modern form by Hohenberg and Kohn, who showed that the ground state of an electron gas in an external potential can be uniquely characterized by the electron density [4], and by Kohn and Sham, who showed how to include the effects of exchange and correlations [5]. The implications of these ideas were soon extended to finite temperatures in the context of the grand canonical framework [6] (see also [7,8,9] and references therein) and to the statistical mechanics of non-uniform classical fluids, such as the liquid–vapor interface [10,11,12,13] (see also [14] for more references).

In previous work, we derived the classical DFT as an application of the method of maximum entropy [15]. A central concept is the use of entropy itself as a tool to generate optimal approximations to probability distributions [16] in terms of those variables that capture the relevant physical information, namely the particle density n(x). We showed that the entropic DFT (eDFT) approach directly leads to Evans’ variational principle of the classical DFT [11].

In this paper, we are concerned with the very foundations of the DFT framework and our main goal is to extend the entropic DFT (eDFT) formalism to the quantum domain. We emphasize that our goal is neither to derive an alternative to DFT nor to develop improvements to the approximations that are inevitably necessary to the successful implementation of DFT in practical applications. Although information theory has been used to quantify chemical concepts [17,18], alternative information-theoretic interpretations of DFT have been suggested [19,20], mostly based on the principle of minimum Fisher information [21]. Our reformulation stands out from three different aspects: First, the maximum entropy foundation on which our formulation is constructed is a completely general method of inference about quantum systems with incomplete information, regardless of the source of the information. In this interpretation of the maximum entropy, information is neither a physical quantity stored in the system, nor is an amount of uncertainty in the probability distribution or the density matrix; it is rather a constraint under which one is to update their degree of rational belief. This is important, especially in the case of DFT. The constraint on the density function arises from a computational assumption that the particle density function of the system is known. However, this information is neither gathered by measurements nor directly obtained from the equilibrium density matrix. Second, we work in the canonical ensemble with a fixed number of particles. This liberates the foundation of DFT from the second quantization. Third and finally, our reformulation is carried out for the more general DFT at finite temperature, which, to our knowledge, had not been tackled by information-theoretic approaches prior to this work.

In Section 2, we review the use of relative entropy as a tool to update density operators in response to new information and we extend the use of entropy as a tool to derive optimal approximations from the classical context [16] to the quantum domain. In Section 3, we construct the entropic DFT formalism and prove a form of the Hohenberg–Kohn theorem at finite temperature within the canonical (fixed number of particles) framework. In Section 4, as an illustration of the eDFT formalism, we discuss the Kohn–Sham model in the local density approximation. Finally, in Section 5, we summarize our conclusions.

## 2. Preliminaries

The realization that a fundamental theory such as thermodynamics should be interpreted as an application of a general scheme for inference on the basis of information codified into constraints can be traced to Brillouin and Jaynes [22,23,24,25,26]. According to Jaynes—as motivated by Shannon’s axioms [27]—entropy is interpreted as the amount of information that is missing in a probability distribution. The preferred probability distribution is that which agrees with what we know—the information codified into the constraints—but is maximally ignorant about everything else. Thus, one is led to maximize the entropy subject to constraints, a procedure dubbed the MaxEnt method.

A drawback of this approach is that the interpretation of entropy as an amount of missing information is not completely satisfactory. To address this problem, Shore and Johnson [28] proposed that one could avoid invoking questionable measures of information by directly axiomatizing the method for updating probabilities through a variational principle that involved maximizing an entropy functional satisfying certain desirable properties. The question of why one should adopt a variational principle was later clarified by Skilling [29], who proposed a simple ranking strategy; in order to select an optimal distribution (he had in mind the more general case of positive additive distributions which include, e.g., intensities in an image), one proceeds by ranking the distributions according to some preference criteria and then choosing the one which ranks the highest. The ranking scheme is naturally implemented by associating a real number—the entropy—to each distribution with the preference criteria fixed through the axioms of Shore and Johnson. In later work, the nature of the method of maximum entropy was further streamlined as a scheme *designed* to update probabilities when confronted with new information. In this approach, the question “what is information?” receives a very simple answer. *Information is just the constraints we decide to impose on our beliefs,* and there is no need to define “amounts” of information. The motivation behind the design criteria was clarified and their number reduced from five to two [30,31,32,33] (reviewed in [34,35]).

### 2.1. The Quantum MaxEnt Method

The task of extending the method of maximum entropy to the quantum domain as a method to update density operators was carried out by Vanslette [33]. The goal is to update a prior density operator $\hat{\sigma }$ when provided with new information in the form of the expected value of some self-adjoint operators $\langle {\hat{A}}_{i}\rangle =A_{i}$. Vanslette showed that the Umegaki relative entropy [36],

(1) $$S_{r}\left[\hat{\rho }|\hat{\sigma }\right]\overset{\mathrm{def}}{=}-\mathrm{Tr}\left(\hat{\rho }\log\hat{\rho }-\hat{\rho }\log\hat{\sigma }\right)\;,$$

provides the *unique* criterion to rank density operators $\hat{\rho }$ relative to the prior $\hat{\sigma }$.

The maximization of $S_{r}\left[\hat{\rho }|\hat{\sigma }\right]$ subject to the constraints $\langle {\hat{A}}_{i}\rangle =A_{i}$ and normalization,

(2) $$\delta \left[S_{r}\left[\hat{\rho }|\hat{\sigma }\right]+\alpha_{0}\left(1-\mathrm{Tr}\hat{\rho }\right)+\sum_{i}\alpha_{i}\left(A_{i}-\mathrm{Tr}\hat{\rho }{\hat{A}}_{i}\right)\right]=0\;,$$

leads to the posterior density operator

(3) $${\hat{\rho }}^{*}=\frac{1}{Z}\exp\left(\log\hat{\sigma }-\sum_{i}\alpha_{i}{\hat{A}}_{i}\right)\;,$$

where

(4) $$Z\left(\alpha_{i}\right)=e^{\alpha_{0}}=\mathrm{Tr}\exp\left(\log\hat{\sigma }-\sum_{i}\alpha_{i}{\hat{A}}_{i}\right)\;.$$

Substituting ${\hat{\rho }}^{*}$ back into Equation (1) gives the value of the maximized entropy,

(5) $$S\left(A_{i}\right)\overset{\mathrm{def}}{=}S_{r}\left[{\hat{\rho }}^{*}|\hat{\sigma }\right]=\sum_{i}\alpha_{i}A_{i}+\log Z\;.$$

It is widely known that the classical MaxEnt method leads to a mathematical formalism characterized by a contact structure (see, e.g., [37,38]). In a parallel development, the use of Legendre transforms in the context of DFT has also been widely explored [7,39,40]. These results can be extended to the quantum domain, leading to a similar contact structure (see, e.g., [41]). The significance of these results is that the physical content of the formalism is preserved under Legendre transformations quite independently of restrictions to thermal equilibrium and of the physical significance of the so-called “free energies” or Massieu functions.

### 2.2. Optimal Approximations of Density Operators

The last prerequisite for the construction of the DFT formalism is a systematic method of approximation for density operators. The method we adopt is an extension of the technique developed by Tseng and Caticha in the classical context [16]. The problem is that the exact probability distributions *Q* obtained using the MaxEnt method are often too intractable to be useful in actual calculations. The solution is to consider a family of more tractable trial distributions $P_{\theta }$ dependent on some parameters θ. The goal is to select the trial distribution $P_{\theta^{*}}$ that best approximates the exact distribution *Q*. In [16], it was argued that the criterion to select the optimal parameters $\theta^{*}$ is again provided by the method of maximum entropy; the optimal $P_{\theta^{*}}$ is that which is “closest” to the exact *Q* in the sense that it maximizes the relative entropy $S[P_{\theta }|Q]$.

Next, we extend this approximation technique to the quantum domain. We consider a family of tractable density operators ${\hat{\rho }}_{\theta }$ parameterized by parameters θ. The member of the trial family ${\hat{\rho }}_{\theta }$ that best approximates the exact density operator ${\hat{\rho }}^{*}$ is the one which maximizes the entropy of ${\hat{\rho }}_{\theta }$ relative to ${\hat{\rho }}^{*}$:

(6) $${\left.\frac{\partial }{\partial \theta }S_{r}\left[{\hat{\rho }}_{\theta }|{\hat{\rho }}^{*}\right]\right|}_{\theta =\theta^{*}}=0\;.$$

As an example, consider the special case where ${\hat{\rho }}^{*}$ and ${\hat{\rho }}_{\theta }$ take the exponential form,

(7) $${\hat{\rho }}^{*}=\frac{1}{Z}e^{-\beta \hat{H}}\;\mathrm{and}\;{\hat{\rho }}_{\theta }=\frac{1}{Z_{\theta }}e^{-\beta {\hat{H}}_{\theta }}\;;$$

The Gibbs inequality,

(8) $$S_{r}\left[{\hat{\rho }}_{\theta }|{\hat{\rho }}^{*}\right]\leq 0\;,$$

reduces to the Bogolyubov inequality,

(9) $$F\leq F_{\theta }+{\langle \hat{H}-{\hat{H}}_{\theta }\rangle }_{\theta }\;,$$

where

(10) $$F=-\frac{1}{\beta }\log Z\;,\;F_{\theta }=-\frac{1}{\beta }\log Z_{\theta }\;,\;\mathrm{and}\;{\langle \cdot \rangle }_{\theta }=\mathrm{Tr}\left[{\hat{\rho }}_{\theta }\left(\cdot \right)\right]\;.$$

Thus, the argument above shows that the popular approximation method based on the Bogolyubov inequality (see, e.g., [42]) is a special case of the more general approximation method based on entropy maximization.

## 3. Density Functional Formalism

The goal of the DFT formalism is to find tractable approximations to study the structure of matter. The first crucial step is to recognize that the quantity that captures the desired structural information is the electron density n(x). We wish to design a formalism in which the central role played by the electron density is explicitly displayed.

In the absence of magnetic fields, the time-independent Schrödinger equation for an electron gas of *N* particles is

(11) $$\hat{H}|\psi \rangle =E|\psi \rangle \;,$$

where

(12) $${\hat{H}}_{v}={\hat{H}}^{\left(0\right)}+\hat{V}=\hat{K}+\hat{U}+\hat{V}=\sum_{i=1}^{N}\frac{{\hat{p}}_{i}^{2}}{2m}+\frac{e^{2}}{2}\sum_{j\neq k}^{N}\frac{1}{|{\hat{x}}_{j}-{\hat{x}}_{k}|}+\sum_{l=1}^{N}v\left({\hat{x}}_{l}\right)\;,$$

and |ψ〉 is an antisymmetrized product of *N* two-spinor orbitals. The potential $\hat{U}$ describes interparticle interactions and the potential $\hat{V}$ describes interactions with nuclei and other external potentials.

### 3.1. Introducing Density as the Relevant Variable

We are interested in the thermal properties of an inhomogeneous electron fluid, and therefore, we need trial states that describe both thermal equilibrium and inhomogeneity. The former is imposed by a constraint on the expected value of energy and the latter is incorporated by constraints on the expected value n(x) of the electron density $\hat{n}\left(x\right)$. The continuous density function n(x) plays a role analogous to the discrete parameters θ in Equations (6)–(10).

Adopting a uniform prior, the relevant trial states are obtained by maximizing the entropy

(13) $$S_{r}\left[\hat{\rho }|\hat{1}\right]=-\mathrm{Tr}\hat{\rho }\log\hat{\rho }\;,$$

subject to the constraints

(14) $$\begin{array}{ccc} \mathrm{Tr}\hat{\rho } & = & 1\;, \end{array}$$

(15) $$\begin{array}{ccc} \mathrm{Tr}\hat{\rho }{\hat{H}}_{v} & = & E\;, \end{array}$$

(16) $$\begin{array}{ccc} \mathrm{and}\;\mathrm{Tr}\hat{\rho }\hat{n}\left(x\right) & = & n(x)\;, \end{array}$$

where

(17) $$\hat{n}\left(x\right)=\sum_{i=1}^{N}\delta \left({\hat{x}}_{i}-x\right)\;\mathrm{and}\;\int d^{3}x\;n\left(x\right)=N\;.$$

To be clear, throughout this work, the trace is taken over the Hilbert space of a fixed number *N* of particles, and in this respect, our formalism *resembles* the canonical ensemble approach. Indeed, all states |ψ〉 in the Hilbert space are eigenstates of the number operator,

(18) $$\hat{N}|\psi \rangle =\int d^{3}x\;\hat{n}\left(x\right)|\psi \rangle =N|\psi \rangle \;\mathrm{so}\;\mathrm{that}\;\langle \psi |\hat{N}|\psi \rangle =N\;,$$

but they need not be eigenstates of the density operators $\hat{n}\left(x\right)$. Our formalism *differs* from the canonical formalism in that Equation (16) represents an additional infinite number of constraints—one constraint on the expected density function n(x) at each point in space. Due to (18), the expected density function n(x) is not arbitrary; it is constrained to obey (18).

Proceeding to the MaxEnt analog of Equation (3), we find the trial density operator

(19) $${\hat{\rho }}_{n}=\frac{1}{Z_{v}}\exp\left(-\beta {\hat{H}}_{v}-\int d^{3}x\;\alpha \left(x\right)\hat{n}\left(x\right)\right)\;,$$

where

(20) $$Z_{v}\left(\beta ;\alpha \right]=\mathrm{Tr}\exp\left(-\beta {\hat{H}}_{v}-\int d^{3}x\;\alpha \left(x\right)\hat{n}\left(x\right)\right)\;,$$

and where β and the infinite number of Lagrange multipliers α(x) are implicitly determined by

(21) $$\frac{\partial \log Z_{v}\left(\beta ;\alpha \right]}{\partial \beta }=-E\;\mathrm{and}\;\frac{\delta \log Z_{v}\left(\beta ;\alpha \right]}{\delta \alpha (x)}=-n\left(x\right)\;,$$

with the additional constraint (17),

(22) $$\int d^{3}x\;n\left(x\right)=-\int d^{3}x\frac{\delta Z_{v}\left(\beta ;\alpha \right]}{\delta \alpha (x)}=N\;.$$

The notation $Z_{v}\left(\beta ;\alpha \right]$ indicates that *Z* is a function of β and a functional of α(x) and depends on v(x) through the Hamiltonian ${\hat{H}}_{v}$. At this point in the argument, there is no implication that the trial states ${\hat{\rho }}_{n}$ are in any way more computationally tractable than the exact state ${\hat{\rho }}^{*}$ obtained from (19) by setting α(x) to zero.

Next, we calculate the entropy of ${\hat{\rho }}_{n}$ relative to the uniform prior to define the trial entropy,

(23) $$S_{r}\left[{\hat{\rho }}_{n}|\hat{1}\right]=\beta E+\int d^{3}x\;\alpha \left(x\right)n\left(x\right)+\log Z_{v}\left(\beta ;\alpha \right]\;\overset{\mathrm{def}}{=}S_{v}\left(E;n\right]\;.$$

An important symmetry of the DFT formalism, which is what makes the whole DFT formalism work, arises from the fact that the dependence of ${\hat{\rho }}_{n}$ and $Z_{v}\left(\beta ;\alpha \right]$ on v(x) and α(x) occurs only through the particular combination

(24) $$\alpha_{\mathrm{int}}\left(x\right)\overset{\mathrm{def}}{=}\alpha \left(x\right)+\beta v\left(x\right)\;.$$

The reason for the subscript ‘int’, which denotes ‘intrinsic’, will become clear later in Equation (56). This DFT symmetry implies that a change in the potential v(x) can be compensated by a suitable change in the multiplier α(x) in such a way that $\alpha_{\mathrm{int}}\left(x\right)$ and the expected density n(x) remain unaffected. From (12) and (24), we find that (20) can be written as

(25) $$Z_{v}\left(\beta ;\alpha \right]=\mathrm{Tr}\exp\left(-\beta {\hat{H}}^{\left(0\right)}-\int d^{3}x\;\alpha_{\mathrm{int}}\left(x\right)\hat{n}\left(x\right)\right)\overset{\mathrm{def}}{=}Z\left(\beta ;\alpha_{\mathrm{int}}\right]\;,$$

so that Equations (19) and (21) become

(26) $${\hat{\rho }}_{n}=\frac{1}{Z(\beta ;\alpha_{\mathrm{int}}]}\exp\left(-\beta {\hat{H}}^{\left(0\right)}-\int d^{3}x\;\alpha_{\mathrm{int}}\left(x\right)\hat{n}\left(x\right)\right)$$

and

(27) $$\;n\left(x\right)=-\frac{\delta \log Z(\beta ;\alpha_{\mathrm{int}}]}{\delta \alpha_{\mathrm{int}}\left(x\right)}\;.$$

### 3.2. The Entropic DFT Variational Principle

The exact canonical density operator ${\hat{\rho }}^{*}$ is found by maximizing (13) subject to (14) and (15). The result can be read off Equation (19) by setting α(x)=0:

(28) $${\hat{\rho }}^{*}=\frac{1}{Z_{v}\left(\beta \right)}\exp\left(-\beta {\hat{H}}_{v}\right)\;\mathrm{and}\;Z_{v}\left(\beta \right)=\mathrm{Tr}\exp\left(-\beta {\hat{H}}_{v}\right)\;.$$

(We use a star * to denote exact canonical quantities.) The goal is to approximate ${\hat{\rho }}^{*}$ by the best matching member of the family $\left\{{\hat{\rho }}_{n}\right\}$ with all density operators referring to the same β and *N*. This involves maximizing the entropy of ${\hat{\rho }}_{n}$ relative to ${\hat{\rho }}^{*}$:

(29) $${\left.\frac{\delta S_{r}\left[{\hat{\rho }}_{n}|{\hat{\rho }}^{*}\right]}{\delta n(x)}\right|}_{\beta ,N}=0\;.$$

From (19) and (28), we find

(30) $$S_{r}\left[{\hat{\rho }}_{n}|{\hat{\rho }}^{*}\right]=\int d^{3}x\;\alpha \left(x\right)n\left(x\right)+\log Z_{v}\left(\beta ;\alpha \right]-\log Z_{v}\left(\beta \right)\;.$$

Introducing a Lagrange multiplier $\alpha^{*}$ to enforce the constraint on *N*, we have

(31) $$\frac{\delta }{\delta n(x)}{\left[S_{r}\left[{\hat{\rho }}_{n}|{\hat{\rho }}^{*}\right]+\alpha^{*}\left(N-\int d^{3}x^{\prime }\;n\left(x^{\prime }\right)\right)\right]}_{\beta }=0\;.$$

From the construction above, one might expect that the optimal ${\hat{\rho }}_{n}$ coincides with the exact ${\hat{\rho }}^{*}$. We can check that this is indeed the case. Substituting Equation (30) into (31), we find

(32) $$\int d^{3}x^{\prime }\;\left[n\left(x^{\prime }\right)+\frac{\delta \log Z_{v}\left(\beta ;\alpha \right]}{\delta \alpha (x^{\prime })}\right]\frac{\delta \alpha (x^{\prime })}{\delta n(x)}=\alpha^{*}-\alpha \left(x\right)$$

The LHS vanishes by Equation (21). Therefore, the optimal ${\hat{\rho }}_{n}$ is achieved for $\alpha \left(x\right)=\alpha^{*}$. From (19), (28) and (30), we see that $\alpha^{*}=0$, which means that imposing the *N* constraint was unnecessary; the optimal density reproduces the exact density $n^{*}\left(x\right)$, whether the variations δn(x) preserve the total *N* or not.

We conclude that the entropic DFT variational principle,

(33) $${\left.\frac{\delta S_{r}\left[{\hat{\rho }}_{n}|{\hat{\rho }}^{*}\right]}{\delta n(x)}\right|}_{n^{*}\left(x\right)}=0\;,$$

leads to an optimal ${\hat{\rho }}_{n}$ which coincides with the exact canonical ${\hat{\rho }}^{*}$ in Equation (28):

(34) $${\hat{\rho }}_{n}^{\mathrm{opt}}={\hat{\rho }}^{*},\;\mathrm{where}\;\alpha^{\mathrm{opt}}\left(x\right)=\alpha^{*}=0\;.$$

Thus, at this point, our “approximation” scheme is (trivially) exact; by explicit construction, we have demonstrated the existence of a functional of the density n(x), β, and *N*—the relative entropy $S_{r}\left[{\hat{\rho }}_{n}|{\hat{\rho }}^{*}\right]$—that assumes its maximum value at the exact density $n^{*}\left(x\right)$. At this point, however, we have not yet shown that this variational principle is equivalent to the thermal DFT principle derived by Mermin [6]. This we show next.

### 3.3. The DFT Theorem

Equations (23) and (30) allow us to write

(35) $$S_{r}\left[{\hat{\rho }}_{n}|{\hat{\rho }}^{*}\right]=-\beta \Omega_{v}\left(\beta ;n\right]-\log Z_{v}\left(\beta \right)$$

where we have introduced the “free energy” functional

(36) $$\Omega_{v}\left(\beta ;n\right]\overset{\mathrm{def}}{=}E-\frac{1}{\beta }S_{v}\left(E;n\right]\;.$$

The new functional $\Omega_{v}$,

(37) $$\Omega_{v}\left(\beta ;n\right]=-\frac{1}{\beta }\int d^{3}x\;\alpha \left(x\right)n\left(x\right)-\frac{1}{\beta }\log Z_{v}\left(\beta ;\alpha \right]\;,$$

allows us to rewrite the entropic variational principle (31) as

(38) $${\left.\frac{\delta \Omega_{v}\left(\beta ;n\right]}{\delta n(x)}\right|}_{n^{*}\left(x\right)}=0\;.$$

The optimal density $n^{*}\left(x\right)$ is found by minimizing $\Omega_{v}\left(\beta ;n\right]$ at fixed β and *N*. Furthermore, from (37), the multipliers α(x) are obtained from

(39) $$\alpha \left(x\right)=-\beta \frac{\delta \Omega_{v}\left(\beta ;n\right]}{\delta n(x)}\;.$$

From Equation (34), $\alpha^{\mathrm{opt}}\left(x\right)=\alpha^{*}=\mathrm{const}$, we obtain

(40) $${\left.\nabla \frac{\delta \Omega_{v}\left(\beta ;n\right]}{\delta n(x)}\right|}_{n^{*}\left(x\right)}=0\;,$$

which has been called the “core integro-differential equation of DFT” [11].

To proceed further, substitute (12), (15), into (36) to find

(41) $$\Omega_{v}\left(\beta ;n\right]={\langle \hat{K}+\hat{U}\rangle }_{{\hat{\rho }}_{n}}+\int d^{3}x\;v\left(x\right)n\left(x\right)-\frac{1}{\beta }S_{v}\left(E;n\right]\;,$$

so that

(42) $$\Omega_{v}\left(\beta ;n\right]=F_{v}\left(\beta ;n\right]+\int d^{3}x\;v\left(x\right)n\left(x\right)\;,$$

where we have introduced

(43) $$F_{v}\left(\beta ;n\right]\overset{\mathrm{def}}{=}{\langle \hat{K}+\hat{U}\rangle }_{{\hat{\rho }}_{n}}-\frac{1}{\beta }S_{v}\left(E;n\right]\;.$$

We are now ready to state the finite temperature DFT theorem.

**Theorem 1** (The Density Functional Theorem)**.** *The density functional $F_{v}\left[n\right]$ is independent of the external potential v(x),*

(44) $${\left.\frac{\delta F_{v}\left(\beta ;n\right]}{\delta v(x)}\right|}_{\beta ,n(x)}=0\;.$$

*This result justifies dropping the index v,*

(45) $$F\left(\beta ;n\right]\overset{\mathrm{def}}{=}F_{v}\left(\beta ;n\right]\;,$$

*and referring to F(β;n] as the intrinsic density functional (The term ‘intrinsic’ indicates that F(β;n] is independent of the external potential v(x)).*

**Proof.**  The crucial observation behind the entropic DFT formalism is that ${\hat{\rho }}_{n}$ and $Z_{v}\left(\beta ;\alpha \right]$ depend on the external potential v(x) and the Lagrange multiplier function α(x) only through the particular combination $\alpha_{\mathrm{int}}\left(x\right)$ defined in (24). Substitute (23)–(25) into (43) to obtain

(46) $$F_{v}\left(\beta ;n\right]=-\frac{1}{\beta }\int d^{3}x\;\alpha_{\mathrm{int}}\left(x\right)n\left(x\right)-\frac{1}{\beta }\log Z\left(\beta ;\alpha_{\mathrm{int}}\right]\;.$$

Then, the derivative $\delta /\delta v(x^{\prime })$ at fixed β and n(x) is

(47) $$\frac{\delta F_{v}\left(\beta ;n\right]}{\delta v(x^{\prime })}=\int d^{3}x^{\prime \prime }\frac{\delta F_{v}\left(\beta ;n\right]}{\delta \alpha_{\mathrm{int}}\left(x^{\prime \prime }\right)}{\left.\frac{\delta \alpha_{\mathrm{int}}\left(x^{\prime \prime }\right)}{\delta v(x^{\prime })}\right|}_{\beta ,n(x)}.$$

Equation (26) shows that keeping n(x) fixed is achieved by keeping $\alpha_{\mathrm{int}}\left(x\right)$ fixed and vice versa; therefore,

(48) $${\left.\frac{\delta \alpha_{\mathrm{int}}\left(x^{\prime \prime }\right)}{\delta v(x^{\prime })}\right|}_{\beta ,n(x)}={\left.\frac{\delta \alpha_{\mathrm{int}}\left(x^{\prime \prime }\right)}{\delta v(x^{\prime })}\right|}_{\beta ,\alpha_{\mathrm{int}}\left(x\right)}=0\;,$$

which implies (44) and concludes the proof. □

Equations (19) and (40) suggest that (up to an additive constant) the multiplier α(x) plays a role *analogous* to that of a chemical potential. Let us then use Equation (39) to introduce

(49) $$\gamma \left(x\right)\overset{\mathrm{def}}{=}-\frac{\alpha (x)}{\beta }=\frac{\delta \Omega_{v}\left(\beta ;n\right]}{\delta n(x)}\;,$$

which we shall call the “*local chemical potential*”. The core Equation (40) has a natural interpretation; the condition for neighboring volume elements to be in equilibrium is that the local chemical potential be uniform:

(50) $${\left.\nabla \gamma (x)\right|}_{n^{*}}=0\;.$$

The optimal value of γ(x) is

(51) $$\gamma^{*}=-\frac{\alpha^{*}}{\beta }=0\;\mathrm{so}\;\mathrm{that}\;\nabla \gamma^{*}=0\;.$$

From Equation (42), we have

(52) $$\delta \Omega_{v}\left(\beta ;n\right]=\delta F\left(\beta ;n\right]+\int d^{3}x\left[n(x)\delta v(x)+v(x)\delta n(x)\right]\;,$$

while Equation (49) gives

(53) $$\delta \Omega_{v}\left(\beta ;n\right]=\int d^{3}x\left(\frac{\delta \Omega_{v}}{\delta v(x)}\delta v\left(x\right)+\frac{\delta \Omega_{v}}{\delta n(x)}\delta n\left(x\right)\right)=\int d^{3}x\left[n(x)\delta v(x)+\gamma (x)\delta n(x)\right]\;.$$

Subtracting these two equations gives

(54) $$\delta F\left(\beta ;n\right]=\int d^{3}x\left[\gamma (x)-v(x)\right]\delta n\left(x\right)\;,$$

which shows that δF/δn can be interpreted as the *local intrinsic chemical potential*,

(55) $$\frac{\delta F(\beta ;n]}{\delta n(x)}\overset{\mathrm{def}}{=}\gamma_{\mathrm{int}}\left(x\right)\;,$$

with

(56) $$\gamma \left(x\right)=\gamma_{\mathrm{int}}\left(x\right)+v\left(x\right)\;\mathrm{and}\;\gamma_{\mathrm{int}}\left(x\right)=-\frac{\alpha_{\mathrm{int}}\left(x\right)}{\beta }\;.$$

Evaluating at $n^{*}$ gives the *equilibrium* intrinsic chemical potential,

(57) $${\left.\frac{\delta F(\beta ;n]}{\delta n(x)}\right|}_{n^{*}}=\gamma_{\mathrm{int}}^{*}\left(x\right)\;.$$

(The term ‘intrinsic’ reminds us that both $\gamma_{\mathrm{int}}\left(x\right)$ and $\gamma_{\mathrm{int}}^{*}\left(x\right)$ are independent of the external potential v(x)).

We mentioned earlier that the multiplier α(x) plays a role *analogous* to that of a chemical potential. We can now be more explicit. Let

(58) $$\mu \left(x\right)=\mu \left(x;n\right]\;\mathrm{and}\;\mu_{\mathrm{int}}\left(x\right)=\mu_{\mathrm{int}}\left(x;n\right]$$

be the actual chemical potential and the intrinsic chemical potential at location *x*,

(59) $$\mu \left(x;n\right]=\mu_{\mathrm{int}}\left(x;n\right]+v\left(x\right)\;.$$

Equilibrium among different volume elements is achieved when

(60) $${\left.\nabla \mu (x;n]\right|}_{n^{*}}=0⟹\mu \left(x;n^{*}\right]=\mu^{*}=\mathrm{const}.$$

Then, Equations (49)–(51) lead us to identify

(61) $$\gamma \left(x\right)=\mu \left(x\right)-\mu^{*}\;\mathrm{so}\;\mathrm{that}\;\gamma^{*}=0\;,$$

and

(62) $$\gamma_{\mathrm{int}}\left(x\right)=\mu_{\mathrm{int}}\left(x;n\right]-\mu \left(x;n^{*}\right]\;.$$

We can express the eDFT variational principle in terms of *F*. Using Equations (38) and (42), we find

(63) $${\left.\frac{\delta }{\delta n(x)}\left(F\left(\beta ;n\right]+\int d^{3}x^{\prime }\;v\left(x^{\prime }\right)n\left(x^{\prime }\right)\right)\right|}_{n^{*}\left(x\right)}=0\;.$$

To summarize, we have reproduced the foundational theorem behind the thermal DFT formalism as an application of maximum entropy methods. This is the main result of this paper. The treatment, so far, has been exact. In the next section, as an illustration of the method, we adapt the well-known Kohn–Sham model to the entropic DFT approach.

## 4. The Kohn–Sham Approximation Scheme

The exact calculation of F(β;n] requires calculating $Z(\beta ;\alpha_{\mathrm{int}}]$. Unfortunately, this is just as difficult as calculating the original canonical partition function $Z_{v}\left(\beta \right)$, which was precisely what we wanted to avoid. An analogous problem arises in the standard many-body theory; even for relatively small particle numbers, the calculation of the *N*-particle wave function becomes impractically difficult because the wave function $\Psi ({\vec{r}}_{1}\ldots {\vec{r}}_{N})$ lives in a 3N-dimensional configuration space. The DFT framework attempts to evade this problem by focusing attention on the hopefully easier problem of calculating the density n(x), which is a *function* that lives in a mere three dimensions. Unfortunately, the problem is not solved, but merely transferred to the calculation of the *functional* F(β;n]. Not all is lost, however, because the reformulation in terms of the density n(x) suggests new useful approximations.

The discussion below parallels closely the ground state formulation of Kohn and Sham [5]. It differs from the grand canonical thermal DFT of Mermin [6] in that here we remain within the canonical framework of fixed particle number. In common with the Hartree–Fock approximation, the Kohn–Sham model reduces an interacting many-particle Schrödinger equation to that of a single particle in the presence of an effective potential that includes exchange and correlation effects. An important advantage is that, unlike Hartree–Fock, the Kohn–Sham framework can in principle be exact. In practice, however, the success of the model hinges on whether the approximations for exchange and correlations are sufficiently simple and accurate. Fortunately, the “local density approximation”, which is exact for a uniform electron gas, and should remain valid for slowly varying potentials, has turned out to be quite successful for the prediction of bond lengths and molecular structures even when these involve inhomogeneities at the atomic scale.

Referring to Equation (43), the idea is that F(β;n] can be split into three terms,

(64) $$F\left(\beta ;n\right]=F_{0}\left(\beta ;n\right]+U_{C}\left[n\right]+F_{\mathrm{xc}}\left(\beta ;n\right]\;.$$

The first term $F_{0}\left(\beta ;n\right]$ represents the intrinsic free energy of a gas of *non-interacting* and *uncorrelated* particles at the same temperature and density. The second term $U_{C}\left[n\right]$ is the classical Coulomb interaction,

(65) $$U_{C}\left[n\right]=\frac{e^{2}}{2}\int d^{3}xd^{3}x^{\prime }\frac{n\left(x\right)n\left(x^{\prime }\right)}{|x-x^{\prime }|}\;,$$

which represents the dominant contribution from the interparticle potential term ${\langle \hat{U}\rangle }_{{\hat{\rho }}_{n}}$ in (43). The third term $F_{\mathrm{xc}}\left(\beta ;n\right]$ is a correction that accounts for all additional exchange and correlations effects. To the extent that we can define $F_{\mathrm{xc}}\left(\beta ;n\right]$ to be the difference

(66) $$F_{\mathrm{xc}}\left(\beta ;n\right]\overset{\mathrm{def}}{=}F\left(\beta ;n\right]-F_{0}\left(\beta ;n\right]-U_{C}\left[n\right]\;,$$

Equation (64) is trivially exact.

We are now ready to substitute (64) into the eDFT variational principle (63). The result is

(67) $${\left[\frac{\delta F_{0}}{\delta n(x)}+v\left(x\right)+\int d^{3}x^{\prime }\frac{e^{2}n\left(x^{\prime }\right)}{|x-x^{\prime }|}+v_{\mathrm{xc}}\left(x;n\right]\right]}_{n^{*}\left(x\right)}=0\;,$$

where we introduced

(68) $$v_{\mathrm{xc}}\left(x;n\right]\overset{\mathrm{def}}{=}\frac{\delta F_{\mathrm{xc}}}{\delta n(x)}\;.$$

So far this is exact. However, to make further progress, we note that although exchange correlations are intrinsically non-local, for a thermal system, we can assume that entanglement effects are appreciable only over short distances. Therefore, it might not be unreasonable to approximate $F_{\mathrm{xc}}$ by a sum over independent volume elements. Accordingly, we adopt the so-called local density approximation,

(69) $$F_{\mathrm{xc}}\left(\beta ;n\right]\approx F_{\mathrm{xc}}^{LDA}\left(\beta ;n\right]=\int d^{3}x\;f_{\mathrm{xc}}\left(n\left(x\right)\right)n\left(x\right)\;,$$

where the function $f_{\mathrm{xc}}\left(n\right)$ is assumed to be known; it is the exchange correlation free energy per particle for a uniform electron gas with density *n*. The corresponding potential

(70) $$v_{\mathrm{xc}}\left(x\right)={\left.\frac{d}{du}\left(f_{\mathrm{xc}}\left(u\right)u\right)\right|}_{u=n(x)}$$

is therefore also known.

To find the optimal density $n^{*}\left(x\right)$ that solves the variational Equation (67), we can use the same trick introduced by Kohn and Sham. They noticed that their variational equation for the ground state—the analogue of our Equation (67)—is exactly of the form one obtains for a gas of non-interacting and uncorrelated particles moving in an effective single-particle potential. This leads us to rewrite (67) as

(71) $${\left[\frac{\delta F_{0}}{\delta n(x)}+v_{\mathrm{eff}}\left(x\right)\right]}_{n^{*}\left(x\right)}=0\;,$$

where

(72) $$v_{\mathrm{eff}}\left(x\right)=v\left(x\right)+\int d^{3}x^{\prime }\frac{e^{2}n\left(x^{\prime }\right)}{|x-x^{\prime }|}+v_{\mathrm{xc}}\left(x\right)\;.$$

Thus, the problem of *N* interacting particles has been translated into the problem of a single particle moving in a density-dependent effective potential created by all the other particles. This shows that we can adopt the same iterative procedure followed with the Hartree self-consistent potential. If $n^{\left(j\right)}\left(x\right)$ is the density at the *j*th iteration, use (72) to construct the potential $v_{\mathrm{eff}}^{\left(j\right)}\left(x\right)$, and solve the single-particle equation,

(73) $$\left[-\frac{1}{2}\nabla^{2}+v_{\mathrm{eff}}^{\left(j\right)}\left(x\right)\right]\psi_{k}^{\left(j\right)}\left(x\right)=\varepsilon_{k}^{\left(j\right)}\psi_{k}^{\left(j\right)}\left(x\right)\;.$$

Then, we construct the density $n^{\left(j+1\right)}\left(x\right)$ for the next iteration as the thermal average,

(74) $$n^{\left(j+1\right)}\left(x\right)=\sum_{k=1}^{k_{\max}}\frac{|\psi_{k}^{\left(j\right)}{\left(x\right)|}^{2}}{1+\exp[\beta \left(\varepsilon_{k}^{\left(j\right)}-\mu \right)]}$$

where the cutoff $k_{\max}$ is such that the occupation of orbitals with $k>k_{\max}$ can be neglected and μ is found by imposing $\int d^{3}x\;n\left(x\right)=N$. The process is repeated until convergence to the optimal $n^{*}$ is achieved.

Just as in the standard Kohn–Sham model, neither the single-particle potential $v_{\mathrm{eff}}\left(x\right)$ nor the wave functions $\psi_{k}$ and energies $\varepsilon_{k}$ are to be given any real physical interpretation. They are auxiliary quantities whose only purpose is the calculation of the physical density $n^{*}\left(x\right)$.

## 5. Conclusions

To summarize our conclusions:

We have produced a *reconstruction* of DFT that makes explicit how DFT fits within an ongoing research program that places the concepts of entropy and information at the very foundation for all of physics (see, e.g., [34]). This includes statistical mechanics [22,23,24,25,26], quantum mechanics [35,43], and as we have shown in this work, also the main techniques to study structure—variational principles including mean field methods and DFT.

We extended the use of entropy as a systematic method to generate optimal approximations from the classical to the quantum domain. This allowed an entropic reconstruction of quantum DFT. This process involves a family of trial density operators parameterized by the particle density. The optimal density operator is found by maximizing the quantum entropy relative to the exact canonical density operator. This approach reproduces the variational principle of DFT and allows a proof of the Hohenberg–Kohn theorem at finite temperature that is simpler in that it evades some of the subtleties of the ground state formalism. Our formalism differs from previous approaches in that (i) the central role of entropy is explicit, and (ii) we remain with the canonical ensemble formalism.

## Data Availability

Data are contained within the article.
